# Evaluation of Capilia TB assay for rapid identification of Mycobacterium tuberculosis complex in BACTEC MGIT 960 and BACTEC 9120 blood cultures

**DOI:** 10.1186/1756-0500-5-44

**Published:** 2012-01-19

**Authors:** Christopher Muchwa, Joseph Akol, Alfred Etwom, Karen Morgan, Patrick Orikiriza, Francis Mumbowa, Paul R Odong, David P Kateete, Kathleen D Eisenach, Moses L Joloba

**Affiliations:** 1 JCRC TB Laboratory, Joint Clinical Research Center, Mengo, Kampala, Uganda; 2 Department of Medical Microbiology, School of Biomedical Sciences, Makerere University College of Health Sciences, Kampala, Uganda; 3 Department of Pathology, University of Arkansas for Medical Sciences, Little Rock, USA

## Abstract

**Background:**

Capilia TB is a simple immunochromatographic assay based on the detection of MPB64 antigen specifically secreted by the *Mycobacterium tuberculosis *complex (MTC). Capilia TB was evaluated for rapid identification of MTC from BACTEC MGIT 960 and BACTEC 9120 systems in Kampala, Uganda. Since most studies have mainly dealt with respiratory samples, the performance of Capilia TB on blood culture samples was also evaluated.

**Methods:**

One thousand samples from pulmonary and disseminated tuberculosis (TB) suspects admitted to the JCRC clinic and the TB wards at Old Mulago hospital in Kampala, Uganda, were cultured in automated BACTEC MGIT 960 and BACTEC 9120 blood culture systems. BACTEC-positive samples were screened for purity by sub-culturing on blood agar plates. Two hundred and fifty three (253) samples with Acid fast bacilli (AFB, 174 BACTEC MGIT 960 and 79 BACTEC 9120 blood cultures) were analyzed for presence of MTC using Capilia TB and in-house PCR assays.

**Results:**

The overall Sensitivity, Specificity, Positive and Negative Predictive values, and Kappa statistic for Capilia TB assay for identification of MTC were 98.4%, 97.6%, 97.7%, 98.4% and 0.96, respectively. Initially, the performance of in-house PCR on BACTEC 9120 blood cultures was poor (Sensitivity, Specificity, PPV, NPV and Kappa statistic of 100%, 29.3%,7%, 100% and 0.04, respectively) but improved upon sub-culturing on solid medium (Middlebrook 7H10) to 100%, 95.6%, 98.2%, 100% and 0.98, respectively. In contrast, the Sensitivity and Specificity of Capilia TB assay was 98.4% and 97.9%, respectively, both with BACTEC blood cultures and Middlebrook 7H10 cultured samples, revealing that Capilia was better than in-house PCR for identification of MTC in blood cultures. Additionally, Capilia TB was cheaper than in-house PCR for individual samples ($2.03 vs. $12.59, respectively), and was easier to perform with a shorter turnaround time (20 min vs. 480 min, respectively).

**Conclusion:**

Capilia TB assay is faster and cheaper than in-house PCR for rapid identification of MTC from BACTEC MGIT 960 and BACTEC 9120 culture systems in real-time testing of AFB positive cultures.

## Background

Genetically related species of the *Mycobacterium tuberculosis *complex (MTC; *M. tuberculosis, M. bovis, M. bovis *BCG, *M. africanum, M. caprae *and *M. cannetti*) cause tuberculosis (TB) [1], a global disease that affects one third of the human population [2,3]. Tuberculosis and HIV form a deadly synergy [4] with approx. 75% of people with HIV/TB co-infection living in sub-Saharan Africa [2,5]. Of the 22 high TB burdened countries, Uganda now ranks 16 with an estimated incidence of 452 cases per 100,000 individuals [3]. Kampala, the capital of Uganda with approx. 2 million people, accounts for ~30% of the nation's TB burden [6].

Accurate diagnosis of TB is crucial for efficient patient management; however, conventional approaches to TB diagnosis still rely on tests with major limitations [7-10]. Smear microscopy, a widely available diagnostic method, has low sensitivity (30% to 60%) especially in patients co-infected with HIV. The chest X-ray, often used as a supplementary test in smear-negative pulmonary TB also has low specificity. Solid culture as a confirmatory test is expensive, lengthy (up to 8 weeks) and is not widely available in resource limited settings [11]. The World Health Organization (WHO) recommends use of liquid cultures in high TB burdened countries due to advantages of rapid detection and incremental yield in comparison with the solid media [12]. However, liquid culture methods are prone to contamination and usually support growth of nontuberculous mycobacteria (NTM), which may as well inhabit the upper respiratory tract and cause disease in immunocompromised patients [13]. This may lead to reporting false results especially during drug sensitivity testing in that NTM are inherently resistant to common anti-TB drugs [14,15]. Further, MTC and NTM cause clinically different clinical symptoms hence prompt identification is crucial for appropriate patient management [16-18].

Recently, nucleic acid amplification tests (NAAT) have been introduced for rapid identification of mycobacteria directly in sample or culture. As such, an in-house PCR assay for identification of MTC was introduced at the Joint Clinical Research Centre (JCRC) in Kampala, Uganda. However, there have been some shortcomings with this method: turnaround time is long (~8 hours) leading to delays in reporting results. Further, we have encountered high rates of false negatives with blood cultures (i.e., templates prepared from BACTEC 9120 system, unpublished observations). Conventional molecular methods are still technologically expensive: reagents require cold storage and shipping; methods are labor-intensive and require separate rooms for DNA extraction, amplification and detection. Batching of samples is usually required for cost effectiveness.

Capilia TB assay is an immunochromatographic method that detects MPB64 protein secreted from MTC bacilli into the culture medium [12]. Originally found in *M. bovis*, similar proteins (i.e. orthologous to MPB64) have been detected in all MTC species and are reportedly rare in NTM. Capilia TB assay is rapid, simple and does not require special equipment [19,20]; it has been found efficient for identification of MTC in South Africa, Thailand and Zambia [20,21]. In this study, the performance of Capilia TB assay was evaluated for rapid detection of MTC from BACTEC MGIT 960 and BACTEC 9120 systems in Kampala, Uganda. Additionally, the performance of Capilia TB on blood cultures was evaluated since previous studies mainly dealt with respiratory samples. The overall Sensitivity, Specificity, PPV and NPV of Capilia TB assay were high and in agreement with values obtained elsewhere.

## Methods

### Study setting and design

Samples for this cross sectional study were obtained from pulmonary and disseminated TB suspects (at baseline, follow up and retreatment cases) admitted to the JCRC clinic and the TB wards at Old Mulago hospital in Kampala, Uganda, from April 2008 to May 2009. Sputum and gastric samples in sterile 50 ml Falcon tubes were obtained directly from TB suspects and injected into sterile 50 ml Falcon tubes while blood samples were drawn intravenously by a study nurse and inoculated into BACTEC 9120 blood culture bottles. Samples were cultured in automated BACTEC MGIT 960 and BACTEC 9120 systems (samples from Old Mulago hospital were transported daily by courier to the JCRC for culture). A convenient sample size (i.e. which did not affect patient management) of 253 AFB-positive blinded samples was considered; these were selected in batches of 30 upon detection of AFB (within 42 days; AFB-negative samples in the same period were excluded). In-house PCRs were performed at the Molecular Biology Laboratory, Makerere University College of Health Sciences (MakCHS) while Capilia TB assays were performed at the JCRC.

### Sample processing and culture

Sputum and gastric samples were processed in Biosafety cabinet class II following the standard N-Acetyl L-cysteine (NALC)/NaOH method [22]. Briefly, an equal volume of decontamination/digestion buffer (6% NaOH, 2.9% Na-citrate and NALC) was added to each sample, vortexed for 5 min and incubated at room temperature for 15 min. The digested samples were neutralized with phosphate buffer (pH 6.8) and mixed thoroughly by inversion. The samples were centrifuged at 3000 g for 15 minutes, and the sediment suspended in 2.5 ml phosphate buffer (pH 6.8). Then, 0.8 ml of the growth supplement, Polymxin B, Amphoterin B, Nalidixic acid, Trimetroprim and Azlocillin (PANTA) was added to the Mycobacterium Growth Indicator Tubes (MGIT), to which 0.5 ml of the digested sample was added and incubated in BACTEC MGIT 960 system for 6 weeks. For blood cultures, the barcodes were scanned prior to incubating directly for 6 weeks in the BACTEC 9120 system. Ziehl Neelsen (ZN) smears were performed on BACTEC MGIT 960 and BACTEC 9120 samples with microbial growth to determine the presence of AFB. Purity checks were performed by sub-culturing on blood agar plates.

### Capilia TB assays

Capilia TB assay (TAUN, Numazu, Japan) was performed on AFB-positive BACTEC samples following the manufacturer's guidelines. For bacterial colonies that grew on solid media (Middlebrook 7H10), a suspension was made by mixing a single colony with 0.2 ml extraction buffer and vortexed; the resultant suspension was then applied onto the Capilia TB strip (following the manufacture's guidelines).

### In-house PCR

The performance of Capilia TB assay was compared with that of in-house PCR, which we used as a baseline identification test for MTC. The in-house PCR protocol was based on the detection of *IS*6110, which is unique to members of the MTC [22]. Templates were prepared from BACTEC cultures and used in a PCR-amplification procedure previously described [22]. Furthermore, MTC were identified to species level using the regions of difference (RD) genotyping method as previously described by Asiimwe et al, 2008 [23]. All the strains belonged to *M. tuberculosis *sensu strict [23].

### Quality control

To avoid cross contamination, separate rooms and equipment were used for sample preparation, DNA extraction and preparation of amplification reactions. In addition, positive and negative controls were always included in the PCRs. Amplification reactions and PCR products were opened in separate UV hoods each located in separate rooms. For cultures, aerosol resistant tips 160 were used and changed for each sample as previously described [22]. Samples with discrepant 161 results were analyzed with the Hains MTB identification kit (Hains life sciences, Nehren, 162 Germany). Blood samples in which AFB grew were further sub-cultured on Middlebrook 7H10 163 agar and analyzed again with both in-house PCR and Capilia TB assay.

### Ethical consideration

Ethical approval was obtained from the institutional review board of the JCRC and the Uganda National Council for Science and Technology. Written informed consent was obtained from all the study participants.

## Results and discussion

### Performance of capilia TB for identification of MTC in BACTEC cultures

In this study, the overall Sensitivity, Specificity, PPV and NPV of Capilia TB assay were high and in agreement with values obtained in other studies [17,20,21,24]. Of the 253 AFB positive samples, 79 (31%, 79/253) were BACTEC 9120 blood cultures while 174 (69%, 174/253) were BACTEC MGIT 960 cultures. M. tuberculosis complex bacilli were identified in 129 samples (51%, 129/253) by Capilia TB and in 130 (51%, 130/253) by in-house PCR. Capilia identified 124 (49%, 124/253) NTM while 130 (51%, 130/253) were identified by in-house PCR. The overall sensitivity, specificity, PPV and NPV of Capilia TB assay were 98.4%, 97.6%, 97.7%, and 180 98.4%, respectively (Table 1). The Kappa statistic was 0.96 indicating almost perfect agreement between the tests.

**Table 1:** Sensitivity and Specificity of Capilia TB in comparison with in-house PCR

	PCR			
**Capilia**		***Positive***	***Negative***	***Total ***
	
	*Positive*	127	2	129
	
	*Negative*	3	121	124
	
	*Total *	130	123	253

Sensitivity (Se) = 98.4%; Specificity (Sp) = 97.58%; Positive predictive value (PPV) = 97.7%; Negative predictive value (NPV) = 98.4% and Kappa statistic (**#**) = 0.96 (almost perfect)

### Performance of capilia TB assay for identification of MTC in pure vs. Contaminated cultures

Of the 253 BACTEC AFB-positive cultures, 139 (55%, 139/253) were contaminated while 114 (45%, 114/253) were pure. Screening contaminated cultures (see Table 2) with Capilia TB assay for MTC revealed Sensitivity, Specificity, PPV and NPV of 95.1%, 99%, 97.5% and 98% respectively, which values were in a similar range with those for pure cultures (i.e., 98.9%, 96.3%, 98.9% and 96.3% respectively). The Kappa statistics for both tests in contaminated and pure samples were 0.98 and 0.95 (almost perfect agreement), respectively.

**Table 2:** Performance of Capilia TB for identification of MTC on contaminated and pure BACTEC cultures

	PCR			
**^a^Capilia**		***Positive ***	***Negative***	***Total ***
	
	*Positive *	39	1	40
	
	*Negative*	2	97	99
	
	*Total *	41	98	139

**^b^Capilia**	*Positive *	86	1	87
	
	*Negative*	1	26	27
	
	*Total *	87	27	114

^a^Performance (%) of Capilia TB on contaminated BACTEC cultures. The Se, Sp, PPV and NPV were 95.1, 99, 97.5 and 98, respectively. ^b^Performance (%) of Capilia TB in pure BACTEC cultures. The Se, Sp, PPV, NPV & (**#**) were 98.9, 96.3, 98.9, 96.3, and (0.98 & 0.95), repectively, (almost perfect agreement)

### Performance of capilia TB in identification of MTC from BACTEC blood cultures and sub-cultures on solid media

Prior to sub-culture on Middle brook 7H10 plates, 57 (72%, 57/79) of the 79 AFB-positive BACTEC 9120 blood cultures were identified as MTC by Capilia TB assay while only four (5%, 4/79) were identified by in-house PCR. Additionally, Capilia TB identified 22 (28%, 22/79) blood cultures as NTM while in-house PCR identified 75 (95%, 75/79). The Sensitivity, Specificity, PPV and NPV of Capilia TB assay on direct blood cultures were 100%, 29.3%, 7% and 100% respectively (Table 3). The Kappa statistic was 0.04, indicating slight agreement between capilia and in-house PCR when used on direct blood culture. Following sub-culturing on Middlebrook 7H10 plates, Capilia TB identified the same number of samples as MTC (i.e. 57/79, 72%) while in-house PCR identified 56 (i.e. 52 more, 71%). When the initial in-house PCR negative blood cultures were tested by the Hains MTB test, 57 (72%, 57/79) samples were confirmed as MTC, which was 100% in agreement with the Capilia TB assay. For in-house PCR, the specificity (29.3% vs. 95.6%) and PPV (7% vs. 98.2%) improved only after sub-culturing on Middlebrook 7H10 plates, whereas values for Capilia TB remained unchanged (Table 3). This confirmed presence of MTC in the 57 blood culture samples as initially identified by Capilia TB assay. The agreement between the two tests was almost perfect (kappa statistic = 0.98). Thus, Capilia TB assay was more efficient than in-house PCR at identifying MTC in BACTEC blood cultures. This demonstrated the superiority of Capilia TB assay to in-house PCR for identification of MTC in various samples. For in-house PCR, the initial high number of false negatives was probably due to PCR inhibitors in blood (such as haeme and/or porphyrins from lysed erythrocytes believed to inhibit Taq polymerase [25]), since PCRs on 7H10 samples turned positive. In terms of materials and labor, the overall cost per sample for identification of MTC with Capilia TB was cheaper than that for in-house PCR (Table 4). Moreover, the cost estimations are more expensive even after excluding equipment (such as thermocyclers).

**Table 3:** Performance of Capilia TB for identification of MTC in direct blood cultures and on solid media (7H10 sub-cultures)

	PCR						
**Capilia**		**Blood culture^a^**			**7H10^b^**		
	
		***Positive ***	***Negative***	***Total ***	***Positive ***	***Negative***	***Total ***
	
	*Positive *	4	53	57	56	1	57
	
	*Negative *	0	22	22	0	22	22
	
	*Total *	4	75	79	56	23	79

^a^The performance (%) of Capilia TB on templates picked directly from blood cultures. The Se, Sp, PPV and NPV were, 100, 29, 7 and 100, respectively. ^b^Performance (%) on Capilia TB assay on Middlebrook 7H10 media. The Se, Sp, PPV, NPV & (**#**) were 100, 95.6, 98.2, 100 and (0.98), respectively (almost perfect agreement)

**Table 4:** Cost per test for performing Capilia TB in comparison with in-house PCR

Materials per test	PCR		Capilia	
	
	*Quantity*	*Cost^a ^*	*Quantity*	*Cost^a^*
*Cryovial*	1	0.34	-	

*Pasteur pipettes*	1	0.19	1	0.19

*Capilia test cassette*	-	-	1	1.84

*PCR reagents*	1	10.00	-	

*Quality control PCR*	1	0.53	-	

*Pipette tips*	8	0.40	-	

*PCR tubes*	1	0.05	-	

***Total cost^a^***		**12.59**		**2.03**

-Not applicable; ^a^US dollars

### Limitations

Due to limited resources, we could not sequence-confirm MTC species nor perform biochemical assays, which are regarded as gold standards for MTC identification. Furthermore, AFB were detected with ZN staining which is not very sensitive, implying that MTC in ZN negative samples were probably missed. Further, mutations in the mpb64 gene usually lead to detection of false negatives [12,24] while NTM testing positive with Capilia TB tests have been recently reported [12,24]; these, coupled with the failure to distinguish members of the MTC and failure to work on clinical specimens somehow negate the efficiency of Capilia TB assays. These shortcomings are also re-iterated in the manufacturer's kit inserts implying that the kit should be cautiously used.

## Conclusion

Capilia TB assay performed better and was cheaper than IS6110 in-house PCR for rapid identification of MTC from BACTEC MGIT 960 and BACTEC 9120 systems. The optimal performance of in-house PCR on blood cultures requires an additional isolation step on solid media for optimum performance.

## Abbreviations

AFB: Acid fast bacilli; MTC: Mycobacterium tuberculosis complex; MTB: Mycobacterium tuberculosis; IS6110: Insertion Sequence 6110; JCRC: Joint Clinical Research Center; NAAT: Nucleic acid amplification tests; MGIT: Mycobacteria growth indicator tube; NTM: Non-tuberculous mycobacteria; PCR: Polymerase chain reaction; TBRU: Tuberculosis Research Unit; ZN: Ziehl-Neelsen; PANTA: Polymxin B, amphoterin B, nalidixic acid, trimetroprim and azlocillin; NPV: Negative predictive value; PPV: Positive predictive value; MakCHS: Makerere University College of Health Sciences.

## Competing interests

The authors declare that they have no competing interests.

## Authors' contributions

CM carried out the experiments and drafted the manuscript, which was proofread and revised by DPK and AE. JA, FM and KM participated in study design; AE performed the statistical analysis. PRO performed the in-house PCR. CM, KE and MLJ conceived the study, which was supervised by KE and MLJ. All authors read and approved the final manuscript.

## References

[B1] Cole ST Comparative and functional genomics of the Mycobacterium tuberculosis complex Microbiology 2002 148 Pt 10 2919 2928 12368425 10.1099/00221287-148-10-2919

[B2] Tsiouris SJ Gandhi NR El-Sadr WM Gerald F Tuberculosis and HIV-Needed A New Paradigm for the Control and Management of Linked Epidemics J Int AIDS Soc 2007 9 3 62 19825140 10.1186/1758-2652-9-3-62 PMC2758896

[B3] World Health Organisation Global tuberculosis control. A short update to the 2009 report http://www.who.int/tb/publications/global_report/2009/update/en/index.html

[B4] The deadly synergy of HIV and tuberculosis Lancet Infect Dis 2010 10 7 441 10.1016/S1473-3099(10)70124-9 20610323

[B5] El-Sadr WM Tsiouris SJ HIV-associated tuberculosis: diagnostic and treatment challenges Semin Respir Crit Care Med 2008 29 5 525 531 10.1055/s-0028-1085703 18810685

[B6] Tuberculosis Profile for Uganda - USAID http://pdf.usaid.gov/pdf_docs/PDACI554.pdf

[B7] Gagneux S Burgos MV DeRiemer K Enciso A MuÃoz S Hopewell PC Small PM Pym AS Impact of Bacterial Genetics on the Transmission of Isoniazid-Resistant Mycobacterium tuberculosis PLoS Pathog 2006 2 6 e61 10.1371/journal.ppat.0020061 16789833 PMC1479046

[B8] Nahid P Pai M Hopewell PC Advances in the diagnosis and treatment of tuberculosis Proc Am Thorac Soc 2006 3 1 103 110 10.1513/pats.200511-119JH 16493157 PMC2658675

[B9] Foulds J O'Brien R New tools for the diagnosis of tuberculosis: the perspective of developing countries Int J Tuberc Lung Dis 1998 3 10 778 783 9783521

[B10] Perkins MD New diagnostic tools for tuberculosis Int J Tuberc Lung Dis 2000 4 12 Suppl 2 S182 S188 11144551

[B11] Reid MJ Shah NS Approaches to tuberculosis screening and diagnosis in people with HIV in resource-limited settings Lancet Infect Dis 2009 9 3 173 184 10.1016/S1473-3099(09)70043-X 19246021

[B12] Shen GH Chiou CS Hu ST Wu KM Chen JH Rapid identification of the Mycobacterium tuberculosis complex by combining the ESAT-6/CFP-10 immunochromatographic assay and smear morphology J Clin Microbiol 49 3 902 907 10.1128/JCM.00592-10 PMC3067747 21159936

[B13] Wagner D Young LS Nontuberculous mycobacterial infections: a clinical review Infection 2004 32 5 257 270 10.1007/s15010-004-4001-4 15624889

[B14] Griffith DE Nontuberculous mycobacterial lung disease Curr Opin Infect Dis 2010 23 2 185 190 10.1097/QCO.0b013e328336ead6 20087178

[B15] Griffith DE Brown-Elliott BA Wallace RJ Jr Diagnosing nontuberculous mycobacterial lung disease A process in evolution. Infect Dis Clin North Am 2002 16 1 235 249 10.1016/S0891-5520(03)00054-0 11917815

[B16] Diagnosis and treatment of disease caused by nontuberculous mycobacteria. This official statement of the American Thoracic Society was approved by the Board of Directors, March 1997. Medical Section of the American Lung Association Am J Respir Crit Care Med 1997 156 2 Pt 2 S1 25 9279284 10.1164/ajrccm.156.2.atsstatement

[B17] Hasegawa N Miura T Ishii K Yamaguchi K Lindner TH Merritt S Matthews JD Siddiqi SH New simple and rapid test for culture confirmation of Mycobacterium tuberculosis complex: a multicenter study J Clin Microbiol 2002 40 3 908 912 10.1128/JCM.40.3.908-912.2002 11880414 PMC120276

[B18] Park MY Kim YJ Hwang SH Kim HH Lee EY Jeong SH Chang CL Evaluation of an immunochromatographic assay kit for rapid identification of Mycobacterium tuberculosis complex in clinical isolates J Clin Microbiol 2009 47 2 481 484 10.1128/JCM.01253-08 19052177 PMC2643687

[B19] Shen GH Chen CH Hung CH Wu KM Lin CF Sun YW Chen JH Combining the Capilia TB assay with smear morphology for the identification of Mycobacterium tuberculosis complex Int J Tuberc Lung Dis 2009 13 3 371 376 19275799

[B20] Ngamlert K Sinthuwattanawibool C McCarthy KD Sohn H Starks A Kanjanamongkolsiri P Anek-vorapong R Tasaneeyapan T Monkongdee P Diem L Diagnostic performance and costs of Capilia TB for Mycobacterium tuberculosis complex identification from broth-based culture in Bangkok, Thailand Trop Med Int Health 2009 14 7 748 753 10.1111/j.1365-3156.2009.02284.x 19392738

[B21] Muyoyeta M de Haas PE Mueller DH van Helden PD Mwenge L Schaap A Kruger C van Pittius NC Lawrence K Beyers N Evaluation of the Capilia TB assay for culture confirmation of Mycobacterium tuberculosis infections in Zambia and South Africa J Clin Microbiol 2010 48 10 3773 3775 10.1128/JCM.01688-09 20686084 PMC2953073

[B22] Muhumuza J Asiimwe BB Kayes S Mugyenyi R Whalen C Mugerwa RD Boom H Eisenach KD Joloba ML Introduction of an in-house PCR for routine identification 322 of M. tuberculosis in a low-income country Int J Tuberc Lung Dis 2006 10 11 1262 1267 17131786

[B23] Asiimwe BB Koivula T Kallenius G Huard RC Ghebremichael S Asiimwe J Joloba ML Mycobacterium tuberculosis Uganda genotype is the predominant cause of TB in Kampala, Uganda Int J Tuberc Lung Dis 2008 12 4 386 391 18371263

[B24] Hirano K Aono A Takahashi M Abe C Mutations including IS6110 insertion in the gene encoding the MPB64 protein of Capilia TB-negative Mycobacterium tuberculosis isolates J Clin Microbiol 2004 42 1 390 392 10.1128/JCM.42.1.390-392.2004 14715787 PMC321697

[B25] Qian Q Tang YW Kolbert CP Torgerson CA Hughes JG Vetter EA Harmsen WS Montgomery SO Cockerill FR Persing DH Direct identification of bacteria from positive blood cultures by amplification and sequencing of the 16S rRNA gene: evaluation of BACTEC 9240 instrument true-positive and false-positive results J Clin Microbiol 2001 39 10 3578 3582 10.1128/JCM.39.10.3578-3582.2001 11574575 PMC88391

